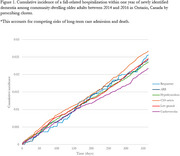# Exploring fall‐related hospitalizations following a new dementia diagnosis using prescribing clusters: a population‐based cohort study in Ontario, Canada

**DOI:** 10.1002/alz70860_103600

**Published:** 2025-12-23

**Authors:** Abby L Emdin, Therese A Stukel, Jennifer Bethell, Laura C Maclagan, Xuesong Wang, Andrea Iaboni, Susan E Bronskill

**Affiliations:** ^1^ Dalla Lana School of Public Health, University of Toronto, Toronto, ON, Canada; ^2^ ICES, Toronto, ON, Canada; ^3^ Institute of Health Policy, Management and Evaluation, University of Toronto, Toronto, ON, Canada; ^4^ University of Toronto, Toronto, ON, Canada; ^5^ KITE Research Institute, Toronto Rehabilitation Institute ‐ University Health Network, Toronto, ON, Canada; ^6^ KITE‐Toronto Rehabilitation Institute, University Health Network, Toronto, ON, Canada; ^7^ Department of Psychiatry, Temerty Faculty of Medicine, University of Toronto, Toronto, ON, Canada; ^8^ Sunnybrook Research Institute, Toronto, ON, Canada

## Abstract

**Background:**

Older adults living with dementia are a heterogenous population which can make studying optimal medication management challenging. Hierarchical clustering, an unsupervised machine learning method, can be used to summarize complex patterns of concurrently prescribed drug therapies across individuals. We aimed to determine if prescribing clusters (derived by grouping individuals dispensed similar medications) were associated with fall‐related hospitalizations in older adults living with dementia.

**Method:**

We identified a cohort of 99,046 older adults (aged ≥67 years) recently diagnosed with dementia in Ontario, Canada between 2014 and 2016 using health administrative data. Individuals were assigned to one of six distinct prescribing clusters: high cardiovascular (angiotensin‐converting enzyme‐specific) (22.6% of the population), central nervous system active (21.3%), hypothyroidism (22.9%), respiratory (3.9%), and angiotensin receptor blocker‐specific cardiovascular (6.1%), and a group with lower dispensation of medications in general (23.1%)). The outcome was fall‐related hospitalizations (emergency department or acute care) over one year following the dementia diagnosis. Cause‐specific survival models estimated the hazard of fall‐related hospitalizations by prescribing cluster, accounting for demographic characteristics, chronic conditions, and history of Beers criteria medications.

**Result:**

Five percent of the cohort experienced a fall‐related hospitalization within the first year of physician‐diagnosed dementia, with highest prevalence in the CNS‐active cluster (5.3%) and the lowest in general low medication use cluster (4.1%). The CNS‐active cluster had a significantly higher relative hazard of fall‐related hospitalization after multivariable adjustment (HR, 1.12, 95% CI: [1.03‐1.22]), compared to those with generally limited medication use.

**Conclusion:**

The hazard of fall‐related hospitalizations differed across prescribing clusters in persons recently diagnosed with dementia, but these differences did not persist after adjustment for key covariates. These methods can be used in future pharmacoepidemiology studies to summarize large amounts of medication data that can then be incorporated into outcomes or causal research.